# Thumb Carpometacarpal Instability: An International Survey on Terminology, Diagnostic Workup, and Treatment

**DOI:** 10.1177/15589447261469600

**Published:** 2026-08-19

**Authors:** Niek J. Nieuwdorp, Maximilian Mayrhofer-Schmid, Caroline A. Hundepool, Leila Harhaus-Waehner, Abhiram R. Bhashyam, Neal C. Chen, J. Michiel Zuidam, Kyle R. Eberlin

**Affiliations:** 1 Harvard Medical School, Boston, MA, USA; 2 Erasmus MC – University Medical Center Rotterdam, the Netherlands; 3 BG Klinikum Unfallkrankenhaus Berlin, Germany; 4 Charité – Universitätsmedizin Berlin, Germany; 5 Xpert Clinics, Amsterdam, the Netherlands

**Keywords:** carpometacarpal joint, diagnostic workup, international survey, ligament reconstruction, thumb carpometacarpal instability

## Abstract

**Background:**

Thumb carpometacarpal (CMC) instability is a controversial topic in hand surgery. This international survey examined the terminology, diagnostic approaches, and treatment strategies used by surgeons worldwide, and explored variations by geographic region and surgical specialty.

**Methods:**

A cross-sectional survey was developed in collaboration with experienced hand surgeons and approved by the research committees of the Federation of European Societies for Surgery of the Hand and the American Association for Hand Surgery. The survey was distributed globally. Responses were analyzed by region and specialty.

**Results:**

Responses were obtained from 208 surgeons across 38 countries. Most preferred the term ‘Thumb CMC instability’ (77%), whereas 16% used ‘Stage I thumb CMC arthritis’. Encounter frequency varied widely, with responses ranging from daily (3%) to rarely (34%). Diagnosis was primarily based on clinical assessment of laxity, supported by radiographs (96%). Nonsurgical management was most commonly selected as first-line treatment (88%), with most surgeons proceeding to surgery only if symptoms persisted (76%). A wide range of surgical techniques was reported, with Eaton-Littler ligament reconstruction used most frequently (32%). U.S. surgeons reported encountering CMC instability more frequently than European surgeons. Choice of surgical technique differed by specialty, with plastic surgeons favoring ligament reconstruction and orthopedic surgeons more often selecting salvage procedures or arthroscopy.

**Conclusions:**

Thumb CMC instability is widely recognized but remains inconsistently defined, diagnosed, and managed. These findings highlight opportunities to improve and standardize care. We advise consistent use of the term ‘thumb CMC instability’ to improve communication in clinical practice and research.

## Introduction

The thumb carpometacarpal (CMC) joint allows for a wide range of motion but offers limited osseous support. Therefore, joint stability depends largely on its surrounding capsuloligamentous structures and stabilizing muscles.^1^ In thumb CMC instability, ligamentous insufficiency and impaired proprioception lead to joint incongruity, resulting in increased joint loading and subsequent synovitis.^2,3^ Clinically, patients present with pain at the thumb base and reduced grip and pinch strength. The condition is most commonly observed in young women with generalized ligamentous laxity and in postmenopausal women.^2,4,5^ If left untreated, persistent joint incongruity may predispose to the development of CMC osteoarthritis.^6-8^

Distinguishing CMC instability from osteoarthritis is clinically consequential, as both conditions require fundamentally different treatment strategies. Instability is managed by restoring joint stability through exercise therapy or ligament reconstruction, whereas osteoarthritis management targets degenerative joint disease and may involve joint-sacrificing procedures such as trapeziectomy.^9,10^ Accurate diagnosis is therefore essential to guide appropriate treatment and avoid exposing patients to interventions that do not address the underlying pathology.

Despite this clinical relevance, many aspects of CMC instability remain poorly standardized. The distinction between instability and early osteoarthritis is often subtle, and instability testing varies considerably among surgeons, in the absence of universally accepted diagnostic criteria.^2,4,9,11,12^ As a result, the conditions are not consistently differentiated in clinical practice.^13^ This uncertainty is reflected in inconsistent terminology, with terms such as ‘early arthritis’ used interchangeably with ‘thumb CMC instability’.^14^ Treatment strategies are equally heterogeneous, with a wide range of nonsurgical and surgical approaches reported.^14,15^ The current evidence base is largely restricted to small case series with short-term follow-up, and clinical guidelines are absent.

This lack of standardization has direct clinical consequences, yet insight into how hand surgeons worldwide currently approach CMC instability remains limited. Understanding current practice is a necessary first step toward guiding future efforts to improve care. The objective of this international survey study was therefore to investigate the terminology, diagnostic approaches, and treatment strategies currently employed by hand surgeons worldwide, and to explore variation across geographical regions and surgical specialties.

## Materials and Methods

### Study Design and Participants

We conducted a cross-sectional survey study to characterize international practice patterns among hand surgeons in the terminology, diagnosis, and management of thumb CMC instability. The study was reviewed and approved by our institutional review board. This study adhered to the Consensus-Based Checklist for Reporting of Survey Studies (CROSS) guidelines.^16^

The electronic survey was distributed in November and December 2025 to national member societies listed by the International Federation of Societies for Surgery of the Hand (IFSSH). Participating societies distributed the survey to their respective members. In addition, the survey was circulated within the Federation of European Societies for Surgery of the Hand (FESSH). All active surgeons involved in the treatment of hand and wrist conditions were eligible to participate. To capture the perspectives of future specialists and broaden the scope of the study, we also invited surgical residents and fellows in training. The survey invitation and link were sent twice, spaced approximately 2 weeks apart.

### Survey Content

The survey was designed by an international panel of experienced hand surgeons to ensure clinical relevance and content validity. It underwent expert review for content and construct validity, followed by pretesting for readability and clarity. The survey instrument was reviewed by the FESSH Research Committee and Council and the American Association for Hand Surgery (AAHS) Research Committee. Following review of the survey content and design, both organizations endorsed the survey and supported its dissemination to their members. All survey responses were collected and securely stored using REDCap, an electronic data capture tool.

**Figure 1.**
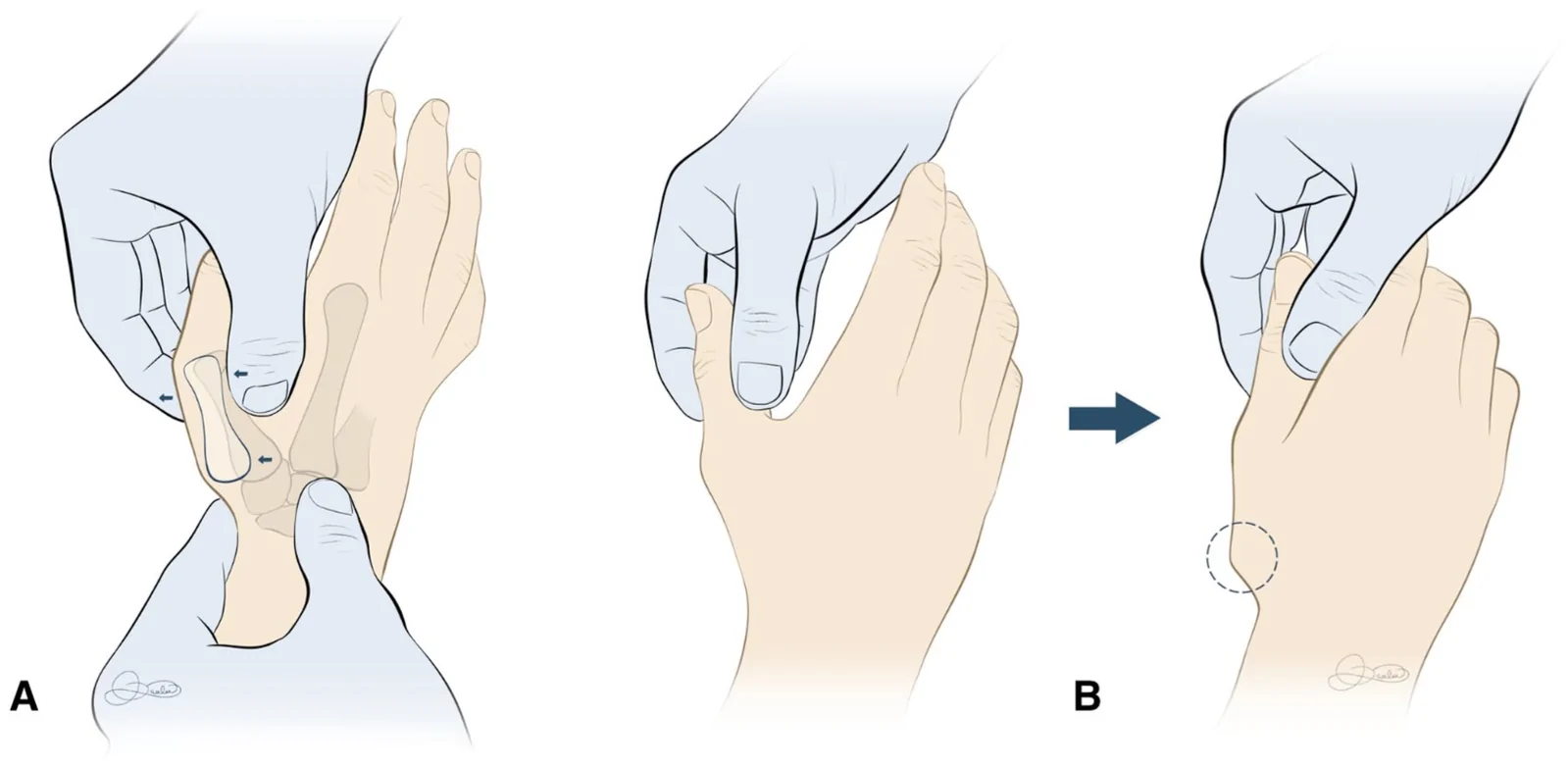
Physical examination findings for thumb carpometacarpal instability. (A) Excessive laxity with dorsoradial displacement of the first metacarpal. (B) Visible subluxation of the first metacarpal on examination.

The survey explored 3 main domains. First, participants were asked about the terminology they use to describe thumb CMC instability and how frequently they encounter such patients, guided by a standardized case vignette:

We present a patient with non-traumatic pain of the thumb CMC joint, with no chondral damage or total joint luxation on X-ray. Physical examination reveals excessive laxity of the CMC joint, particularly during dorsoradial displacement (Figure 1A). The patient presents with no other joint instabilities.

Second, questions addressed diagnostic approaches, including physical examination findings and imaging modalities routinely used, such as plain radiography, computed tomography, and magnetic resonance imaging.

Third, respondents reported their treatment strategies and patient selection criteria, indicating their preferred nonsurgical and surgical approaches and whether their terminology or management would change in the presence of multiple joint instabilities (eg, generalized ligamentous laxity). The complete survey is provided in Supplemental Material S1.

In addition, demographic information was collected, including participants’ age, gender, primary specialty, current training level, practice setting, country of practice, and whether they held a subspecialty certificate in hand surgery.

### Statistical Analysis

Survey outcomes are presented descriptively. Data distribution was assessed using histograms, quantile-quantile plots, and the Shapiro-Wilk test. Normally distributed data are reported as the mean and standard deviation (SD), and non-normally distributed data as the median and interquartile range.

As a secondary analysis, differences between geographic regions and surgical specialties were evaluated using Fisher exact tests and χ^2^ tests, as appropriate. Comparative analyses were restricted to surgeons from the United States and Europe, and to orthopedic and plastic surgeons, as respondents from other regions and specialties were too few in number to permit meaningful subgroup analyses. All respondents were included in the descriptive analyses, whereas only these subgroups were included in the comparative analyses.

All analyses were performed using R statistical software (version 4.5.1; R Foundation for Statistical Computing, Vienna, Austria). A *P* value less than .05 was considered statistically significant.

## Results

**Figure 2.**
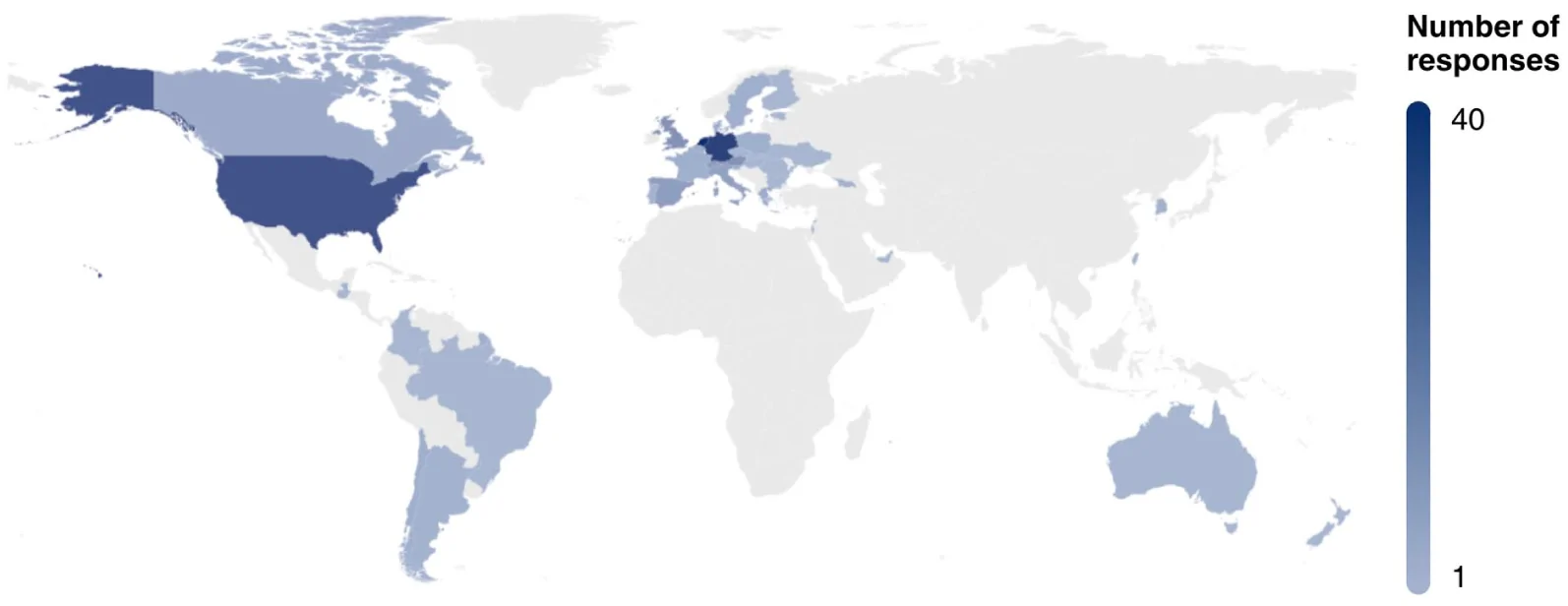
Geographic distribution of survey responses by country.

The survey was distributed through the FESSH and 18 national member societies of the IFSSH, yielding a total of 208 responses. Most respondents were male (72%). Orthopedic surgery was the most common training background (46%), followed by plastic surgery (39%). A detailed overview of respondent characteristics is shown in Table 1. Responses were received from 38 countries (Figure 2).

**Table 1.** Demographic Characteristics of the Participating Surgeons.

Variable	Surgeon characteristics (N = 208)
Age, y, mean (SD)	51 (12)
Sex, No. (%)	
Male	150 (72)
Female	54 (26)
Prefer not to answer	4 (2)
Specialty, No. (%)	
Orthopedic surgery	96 (46)
Plastic surgery	81 (39)
General trauma surgery	19 (9)
Hand surgery	11 (5)
General surgery	1 (0)
Practice setting, No. (%)	
Academic medical center	72 (35)
Teaching hospital	61 (29)
Nonteaching hospital-based employment	21 (10)
Private clinic	48 (23)
Other	6 (3)
Subspecialty hand surgery certification, No. (%)	
FESSH (Europe)	79 (38)
Subspecialty Certificate in Hand Surgery (United States)	29 (14)
National hand surgery certification	43 (21)
No hand surgery certification	57 (27)
Current training level, No. (%)	
Fully qualified surgeon	198 (95)
Resident or fellow	10 (5)
Years as surgeon, y, median [IQR]	15 [8-26]
Years in residency or fellowship, y, median [IQR]	6 [5-11]

Abbreviations: FESSH, Federation of European Societies for Surgery of the Hand; IQR, interquartile range; N, number of surgeons; SD, standard deviation s; y, years.

### Terminology and Frequency of Encounters

**Figure 3.**
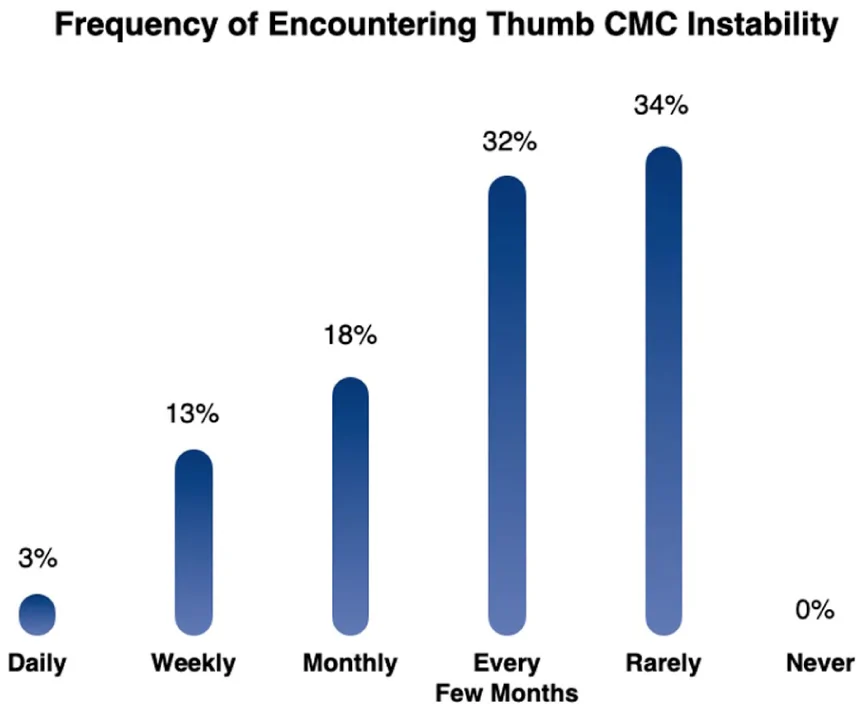
Reported frequency of encountering thumb carpometacarpal instability among surgeons.

The most frequently used diagnostic term was ‘Thumb CMC instability’ (77%), followed by ‘Eaton and Glickel stage I thumb CMC arthritis’ (16%). An overview of all reported diagnostic terms is presented in Table 2. Most respondents considered the diagnosis a precursor to thumb CMC osteoarthritis (71%), whereas 29% regarded it as a standalone condition. A wide variation was observed in the reported frequency of encountering CMC instability, with responses ranging from daily (3%) to rarely (34%) (Figure 3).

**Table 2.** Terminology Used by Surgeons to Describe Patients With Nontraumatic Thumb Carpometacarpal Pain With Laxity.

Term	Number of surgeons No. (%)
Thumb CMC instability	161 (77)
Stage I thumb CMC arthritis^a^	33 (16)
Early-stage thumb CMC arthritis	26 (13)
Thumb CMC (hyper)laxity	4 (2)
Dysplasia of the thumb CMC joint	1 (0)
Proprioceptive deficit of the thumb CMC joint	1 (0)

Abbreviation: CMC, carpometacarpal joint.

Terminology for patients with nontraumatic thumb carpometacarpal pain and clinical joint laxity, without radiographic chondral damage or joint dislocation.

a Stage according to Eaton and Glickel (1987).^17^

### Diagnostic Workup

The most frequently used physical examination test was excessive laxity on dorsoradial displacement of the first metacarpal (Figure 1A) (72%), followed by subluxation upon manipulation (Figure 1B) (64%) and pain on dorsoradial displacement (Figure 1A) (63%). Tenderness on palpation was used by 55% of respondents. When surgeons reported that they tested for excessive laxity on dorsoradial displacement, nearly all (99%) declared that they compared it to the contralateral side.

X-ray was the most commonly used diagnostic modality, with nearly all surgeons (96%) reporting its use. In total, 90% of all surgeons used x-rays to rule out chondral damage, whereas 44% used them to assess joint instability. Magnetic resonance imaging was the second most commonly used modality (36%), primarily to evaluate chondral damage (28%). A complete overview of diagnostic tools is presented in Supplemental Table S2.

### Treatment and Patient Selection

Most surgeons selected nonsurgical management as the initial treatment option (88%). A smaller proportion favored primary surgical intervention (7%), whereas only a minority recommended no treatment (5%). Among respondents opting for nonsurgical management, there was considerable variability in the treatment strategies employed. The most frequently selected modalities were orthotic devices (thumb spica splint) (77%) and physical therapy (67%). In addition, 37% reported prescribing analgesics, and 21% administered corticosteroid injections.

**Figure 4.**
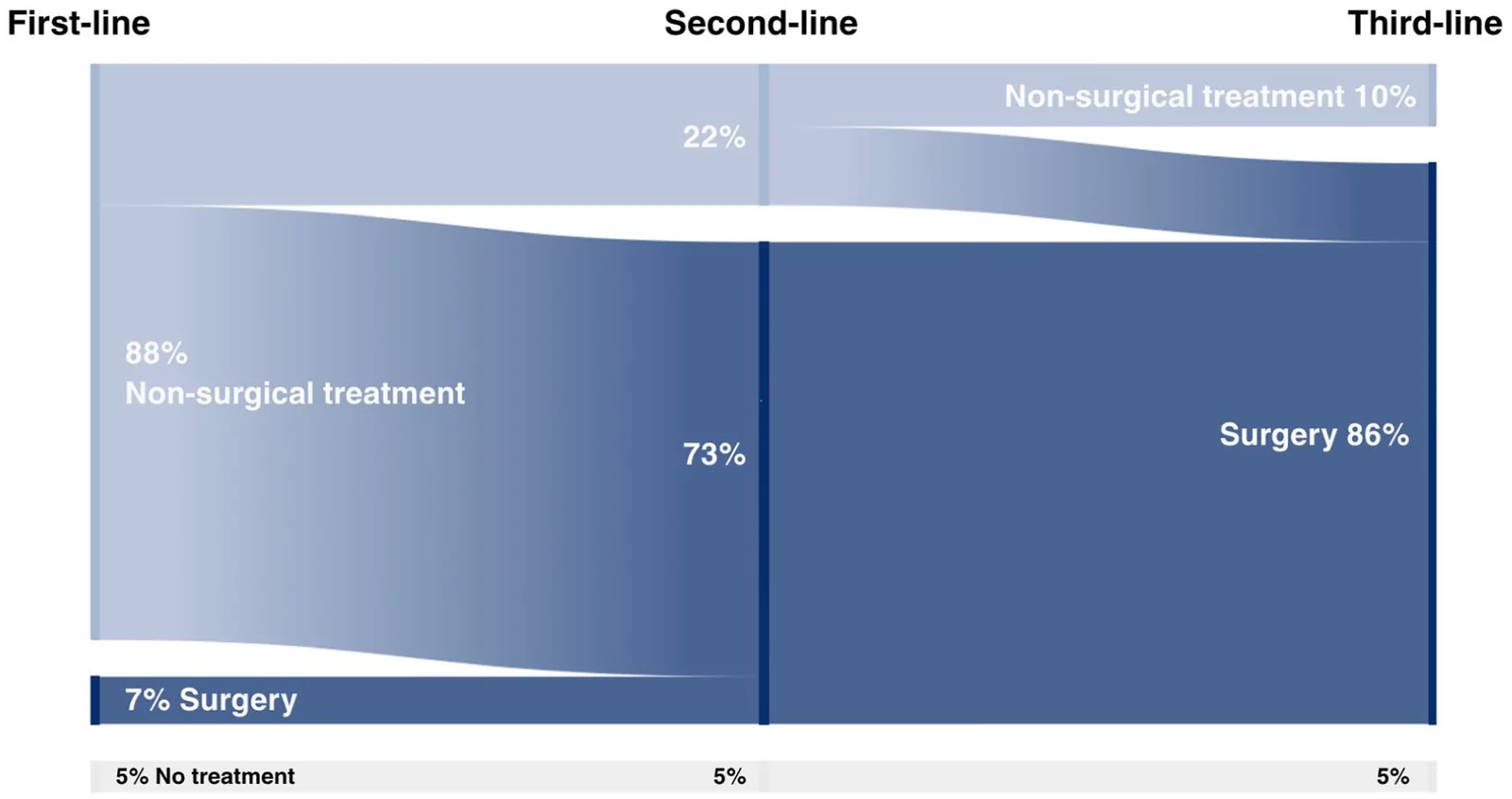
Treatment pathways for thumb carpometacarpal instability. Nonsurgical management was most commonly selected as first-line treatment. After failure, most surgeons proceeded to surgery, whereas a minority continued nonsurgical management.

Of the surgeons who selected nonsurgical management as the initial treatment, 76% indicated they would proceed with surgery if these measures proved ineffective, whereas 24% would continue with nonsurgical management. Figure 4 illustrates the variation in treatment protocols among surgeons. When asked which factors influenced their decision not to recommend surgical intervention, 62% cited the severity of symptoms, 54% considered patient age, 48% the patient’s activity level, and 39% the presence of chondral damage on radiographs. A complete overview of all reported factors is provided in Supplemental Table S3.

As a first-line surgical approach, procedures aimed at improving or reconstructing the affected ligaments were most commonly selected (78%). The most frequently used technique was the Eaton-Littler ligament reconstruction (32%), followed by dorsoradial capsulodesis (22%) and other ligament reconstruction techniques (14%).

**Figure 5.**
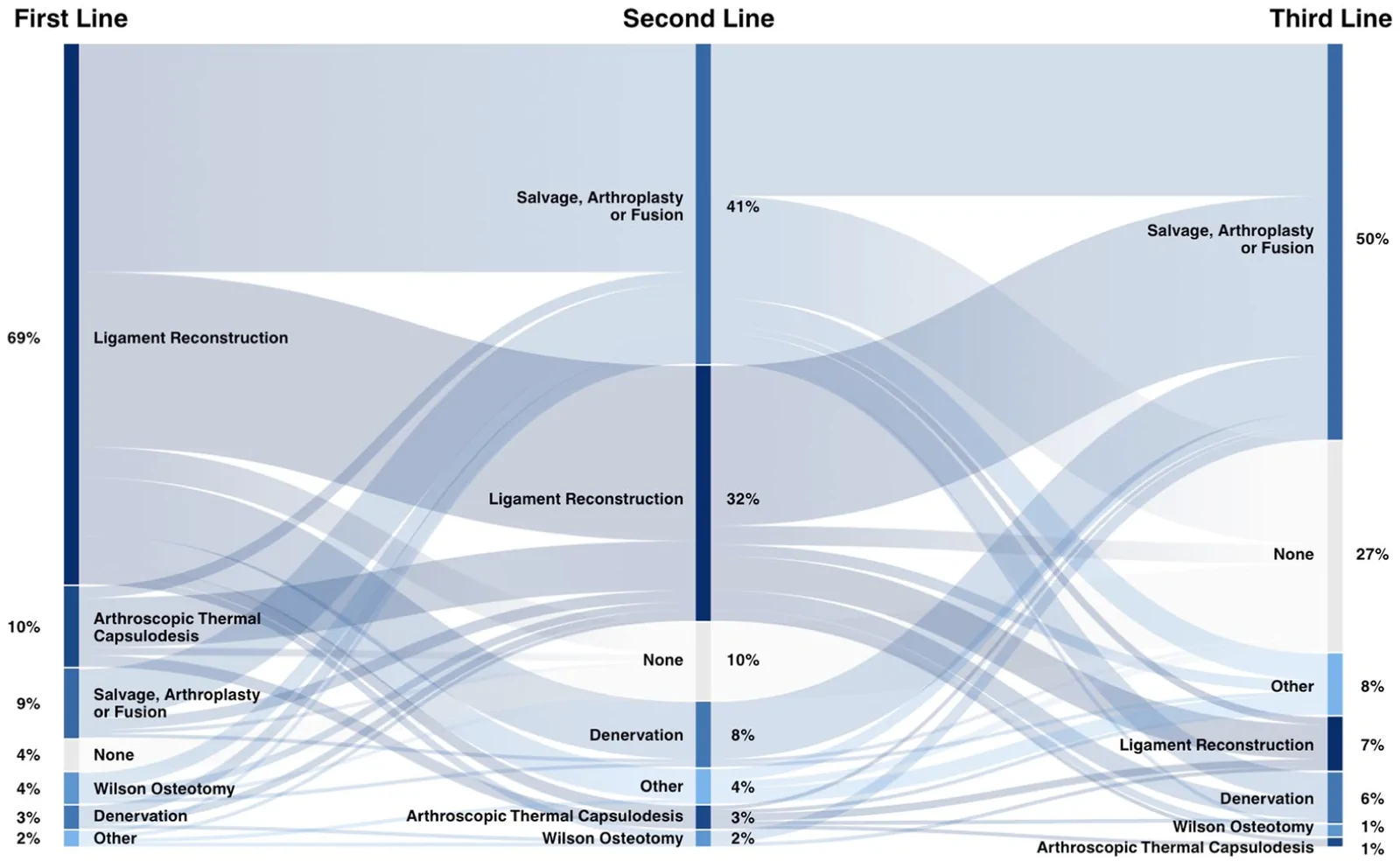
Surgical techniques used for thumb carpometacarpal (CMC) instability, including second- and third-line procedures after failure of initial surgery. *Ligament reconstruction* includes Eaton-Littler reconstruction, dorsoradial capsulodesis, and other ligament reconstruction techniques. *Salvage*, *arthroplasty*, *or fusion* includes trapeziectomy ± ligament reconstruction and tendon interposition, CMC prosthesis, and arthrodesis.

As second- or third-line options after failure of the initial procedure, surgeons more often selected more definitive surgical treatments, such as trapeziectomy with or without ligament reconstruction, prosthesis, or arthrodesis (Figure 5). An overview of all reported surgical techniques indicated as first-, second-, and third-line treatment is presented in Supplemental Material S4.

When determining the appropriate surgical technique, surgeons most frequently reported pain severity (76%) as a key factor influencing their choice, followed by the severity of instability (69%) and patient-related characteristics such as age and comorbidities (69%). A complete overview of all factors associated with surgical technique selection is provided in Supplemental Table S5.

### Multiple Joint Instabilities

When asked whether surgeons would alter their terminology or treatment approach in patients with generalized ligamentous laxity or hypermobility syndromes, 56% of respondents answered ‘Yes’, whereas 44% reported no change to their standard approach. Respondents who reported changing their approach most frequently cited a greater emphasis on nonsurgical management and increased reluctance toward surgical intervention (53%). When surgery was considered, there was a shift toward techniques that did not rely on soft tissues, such as arthrodesis (38%).

### Geographic Differences

Due to the distribution of responses, comparative analyses were restricted to surgeons from the United States (n = 30) and Europe (n = 156). Between regions, there were no significant differences between terminology (*P* = .999) and views on CMC instability as a precursor to osteoarthritis (*P* = .505). Surgeons from the United States reported encountering CMC instability more frequently (*P* = .032) than European surgeons (Supplemental Material S6). No regional differences were found in initial treatment strategy (*P* = .144), conversion to surgery after nonsurgical management (*P* = .197), or initial choice of surgical technique (*P* = .114).

### Specialty Differences

Analyses were limited to orthopedic surgeons (n = 96) and plastic surgeons (n = 81), given the available subgroup sizes. No significant differences were observed between specialties in terminology (*P* = .127), consideration of CMC instability as a precursor to osteoarthritis (*P* = .682), frequency of encountering CMC instability (*P* = .325), initial treatment strategy (*P* = .826), or conversion to surgery after nonsurgical management (*P* = .632). However, the initial choice of surgical technique differed significantly (*P* = .001) between orthopedic and plastic surgeons. Plastic surgeons more often favored ligament reconstruction (83% vs 59%), whereas orthopedic surgeons more frequently selected salvage procedures (12% vs 6%) or arthroscopy (17% vs 1%) (Supplemental Material S7).

## Discussion

Many aspects of thumb CMC instability remain controversial, and the current literature offers little description of its management in clinical practice. This international survey provides insight into how hand surgeons worldwide currently define, diagnose, and manage this condition. In patients with nontraumatic thumb CMC pain and laxity, without chondral damage or complete joint dislocation, most respondents used the term ‘Thumb CMC instability’. Diagnosis was primarily clinical, based on assessment of excessive laxity, and supported by radiographic imaging. Nonoperative treatment was the most common first-line management, with conversion to surgery if treatment failed. When surgery was performed, the Eaton-Littler ligament reconstruction was the most frequently selected procedure.

There was relative consensus on the use of the term ‘Thumb CMC instability’ (77%), which is notable given the heterogeneity in terminology reported in the literature.^14^ Consistent terminology is essential for clear communication and comparability of research. Based on these findings, we support the use of ‘Thumb CMC instability’ as the preferred term for patients with nontraumatic thumb CMC pain and laxity, in the absence of chondral damage or complete joint dislocation on imaging.

Only one-third of respondents (29%) considered CMC instability to be a distinct clinical entity. This is noteworthy, as most surgeons favored procedures aimed at restoring ligamentous stability rather than addressing degenerative joint changes (78%). This suggests that in clinical practice, the 2 conditions are often managed as distinct entities. Available outcome data support this approach, with relatively low rates of progression to osteoarthritis following targeted treatment for instability.^6,10,18-20^ Taken together, the preference for stabilizing interventions and the low observed rate of subsequent progression to osteoarthritis support the continued recognition of CMC instability as a distinct clinical entity.

Wide variation was observed in the frequency of encountering CMC instability, with higher reported rates in the United States compared with Europe, although substantial variability persisted within regions. The underlying reasons remain unclear and may relate to differences in case mix, diagnostic thresholds, or surgical training. Treatment strategies were similarly heterogeneous, including variation in treatment algorithms and surgical techniques. This likely reflects limited evidence and the absence of clear guidelines.^21^ With the current literature largely restricted to small case series and short-term follow-up, management remains controversial and often driven by surgeon preference. These findings highlight the need for standardized, evidence-based diagnostic and treatment frameworks.

Splinting was the most commonly selected nonsurgical treatment (77%), followed by exercise therapy (67%). This contrasts with prior literature emphasizing exercise therapy as the cornerstone of nonsurgical management because of its role in improving active joint stability.^15^ While splinting may provide short-term symptom relief, prolonged use may reduce muscular support and impair active stabilization. These differences highlight the need for comparative studies evaluating the effectiveness of splinting and exercise therapy.

The wide variety of surgical techniques further underscores the limited and inconclusive evidence base. Eaton-Littler ligament reconstruction was the most frequently selected procedure and remains the most extensively studied technique.^6,14,18,19,22,23^ Dorsoradial capsulodesis was also commonly used, despite limited comparative clinical evidence.^14,24,25^ Although cadaveric studies suggest that capsulodesis provides less stability than ligament reconstruction, the clinical relevance of this difference remains uncertain.^26^ Surgical preferences differed between specialties, with plastic surgeons more often favoring ligament reconstruction and orthopedic surgeons more frequently selecting salvage procedures or arthroscopy, likely reflecting differences in training and practice patterns. These findings emphasize the ongoing uncertainty regarding optimal surgical management.

This study has limitations. A response rate could not be calculated, as the survey was disseminated through multiple channels, including society networks and social media, and the total number of surgeons reached across all distribution channels was unknown. Nonresponse bias therefore cannot be excluded, and the representativeness of the sample cannot be fully determined. However, given the exploratory aim of this study, these limitations are unlikely to substantially affect the findings. In addition, the broad dissemination strategy enabled a wide international reach and a substantial number of responses. As such, this study provides a contemporary overview of clinical practice, identifying both areas of consensus and substantial variation. The findings are hypothesis-generating and help define priorities for future research.

Given the substantial heterogeneity identified in this survey, future research should focus on establishing a standardized diagnostic framework for thumb CMC instability. An international Delphi consensus process could be used to systematically identify, refine, and prioritize candidate diagnostic criteria.^27^ In addition, robust prospective outcomes data and comparative effectiveness studies are needed to guide treatment decisions. Such efforts may ultimately support more consistent diagnosis and treatment of thumb CMC instability.

## Conclusions

Thumb CMC instability is widely recognized but remains inconsistently defined, diagnosed, and managed. Most surgeons prefer the term ‘Thumb CMC instability’, supporting its use as the standard terminology in clinical practice and research. The marked variation in encounter rates, diagnostic approaches, and treatment strategies reflects the absence of high-quality evidence and clear guidelines. These findings highlight the need for standardized, evidence-based diagnostic and treatment frameworks.

## Supplemental Material

sj-pdf-1-han-10.1177_15589447261469600 – Supplemental material for Thumb Carpometacarpal Instability: An International Survey on Terminology, Diagnostic Workup, and Treatment Supplemental material, sj-pdf-1-han-10.1177_15589447261469600 for Thumb Carpometacarpal Instability: An International Survey on Terminology, Diagnostic Workup, and Treatment by Niek J. Nieuwdorp, Maximilian Mayrhofer-Schmid, Caroline A. Hundepool, Leila Harhaus-Waehner, Abhiram R. Bhashyam, Neal C. Chen, J. Michiel Zuidam and Kyle R. Eberlin in HAND

sj-pdf-2-han-10.1177_15589447261469600 – Supplemental material for Thumb Carpometacarpal Instability: An International Survey on Terminology, Diagnostic Workup, and Treatment Supplemental material, sj-pdf-2-han-10.1177_15589447261469600 for Thumb Carpometacarpal Instability: An International Survey on Terminology, Diagnostic Workup, and Treatment by Niek J. Nieuwdorp, Maximilian Mayrhofer-Schmid, Caroline A. Hundepool, Leila Harhaus-Waehner, Abhiram R. Bhashyam, Neal C. Chen, J. Michiel Zuidam and Kyle R. Eberlin in HAND

sj-pdf-3-han-10.1177_15589447261469600 – Supplemental material for Thumb Carpometacarpal Instability: An International Survey on Terminology, Diagnostic Workup, and Treatment Supplemental material, sj-pdf-3-han-10.1177_15589447261469600 for Thumb Carpometacarpal Instability: An International Survey on Terminology, Diagnostic Workup, and Treatment by Niek J. Nieuwdorp, Maximilian Mayrhofer-Schmid, Caroline A. Hundepool, Leila Harhaus-Waehner, Abhiram R. Bhashyam, Neal C. Chen, J. Michiel Zuidam and Kyle R. Eberlin in HAND

sj-pdf-4-han-10.1177_15589447261469600 – Supplemental material for Thumb Carpometacarpal Instability: An International Survey on Terminology, Diagnostic Workup, and Treatment Supplemental material, sj-pdf-4-han-10.1177_15589447261469600 for Thumb Carpometacarpal Instability: An International Survey on Terminology, Diagnostic Workup, and Treatment by Niek J. Nieuwdorp, Maximilian Mayrhofer-Schmid, Caroline A. Hundepool, Leila Harhaus-Waehner, Abhiram R. Bhashyam, Neal C. Chen, J. Michiel Zuidam and Kyle R. Eberlin in HAND

sj-pdf-5-han-10.1177_15589447261469600 – Supplemental material for Thumb Carpometacarpal Instability: An International Survey on Terminology, Diagnostic Workup, and Treatment Supplemental material, sj-pdf-5-han-10.1177_15589447261469600 for Thumb Carpometacarpal Instability: An International Survey on Terminology, Diagnostic Workup, and Treatment by Niek J. Nieuwdorp, Maximilian Mayrhofer-Schmid, Caroline A. Hundepool, Leila Harhaus-Waehner, Abhiram R. Bhashyam, Neal C. Chen, J. Michiel Zuidam and Kyle R. Eberlin in HAND

sj-pdf-6-han-10.1177_15589447261469600 – Supplemental material for Thumb Carpometacarpal Instability: An International Survey on Terminology, Diagnostic Workup, and Treatment Supplemental material, sj-pdf-6-han-10.1177_15589447261469600 for Thumb Carpometacarpal Instability: An International Survey on Terminology, Diagnostic Workup, and Treatment by Niek J. Nieuwdorp, Maximilian Mayrhofer-Schmid, Caroline A. Hundepool, Leila Harhaus-Waehner, Abhiram R. Bhashyam, Neal C. Chen, J. Michiel Zuidam and Kyle R. Eberlin in HAND

sj-pdf-7-han-10.1177_15589447261469600 – Supplemental material for Thumb Carpometacarpal Instability: An International Survey on Terminology, Diagnostic Workup, and Treatment Supplemental material, sj-pdf-7-han-10.1177_15589447261469600 for Thumb Carpometacarpal Instability: An International Survey on Terminology, Diagnostic Workup, and Treatment by Niek J. Nieuwdorp, Maximilian Mayrhofer-Schmid, Caroline A. Hundepool, Leila Harhaus-Waehner, Abhiram R. Bhashyam, Neal C. Chen, J. Michiel Zuidam and Kyle R. Eberlin in HAND

sj-pdf-8-han-10.1177_15589447261469600 – Supplemental material for Thumb Carpometacarpal Instability: An International Survey on Terminology, Diagnostic Workup, and Treatment Supplemental material, sj-pdf-8-han-10.1177_15589447261469600 for Thumb Carpometacarpal Instability: An International Survey on Terminology, Diagnostic Workup, and Treatment by Niek J. Nieuwdorp, Maximilian Mayrhofer-Schmid, Caroline A. Hundepool, Leila Harhaus-Waehner, Abhiram R. Bhashyam, Neal C. Chen, J. Michiel Zuidam and Kyle R. Eberlin in HAND
