# Supplementary material for: Thumb Carpometacarpal Instability: An International Survey on Terminology, Diagnostic Workup, and Treatment
Source: Hand (N Y). 2026 Aug 19:15589447261469600. Online ahead of print. doi: 10.1177/15589447261469600 (PMC13490322; doi:10.1177/15589447261469600)
Supplement: Supplementary file 1 [file sj-pdf-1-han-10.1177_15589447261469600.pdf]

Supplemental Material S1:

Survey on terminology, diagnosis, and management of thumb CMC instability.

Introduction:

Thank you for participating in this survey.

In 1973, Eaton and Littler described patients with idiopathic painful hypermobility of the thumb basal joint.[1] This article has withstood the test of time, but in the current literature, many different terms are proposed for this condition, leading to potential communication challenges, and hindering research comparability. The existing literature suggests using the terms 'laxity' or 'hypermobility' to describe the condition of loose ligaments in a joint. When laxity leads to symptoms, it is referred to as 'instability'.[2]

Moreover, there is a lack of consensus on the preferred diagnostic approach for patients with this condition. Additionally, the optimal management remains uncertain, with numerous surgical techniques described in the literature, each with limited supporting evidence.[3]

Your participation in this survey is invaluable as we seek insights from surgeons regarding different aspects of this condition. We aim to gather information on terminology, diagnostic methods, treatment approaches, and surgical preferences. The survey only takes 6 minutes to complete, and your contribution will greatly help in advancing our understanding and improving the management of this condition. Thank you for taking the time to share your expertise and experiences with us.

1. Questions about the Surgeon:

The following questions pertain you and your practice.

What is your age?
Answer: [NUMBER]

What is your gender?
- o Male
- o Female
- o Prefer not to answer

What is your specialty?
- o Orthopedic surgery
- o Plastic surgery
- o General trauma surgery
- o Other (please specify): _______________

In what setting is your primary hand surgery practice?
- o Academic medical center (includes a medical school)
- o Teaching hospital (hospital with trainees but does not include a medical school)
- o Non-teaching hospital-based employment
- o Private clinic (i.e., self-employed or small group practice)
- o Other (please specify): _______________

In what country do you practice? Please add the state if you work in the United States of America.
Answer: [FREE TEXT]

In which country did you complete the majority of your residency training? Please add the state if you work in the United States of America.
Answer: [FREE TEXT]

Have you completed subspecialty hand surgery certification?
- FESSH (Europe)
- Subspecialty Certificate in Hand Surgery (United States)
- No hand surgery certification
- Other (please specify): _______________

What is your current training level?
- Resident
- Fellow
- Fully qualified surgeon

[if the participant chose Resident or Fellow in the previous question]
How many years have you been in residency/fellowship (including your current year)?
Answer: [NUMBER]

[if the participant chose Fully qualified surgeon in the previous question]
How many years have you been a practicing surgeon (excluding residency, including fellowships)? Please round up to the nearest year.
Answer: [NUMBER]

2. Terminology:
We present a patient with non-traumatic pain of the thumb carpometacarpal (CMC) joint, with no chondral damage or total joint luxation on X-ray. Physical examination reveals excessive laxity of the CMC joint, particularly during dorsoradial displacement (see figure 1). The patient presents with no other joint instabilities.

Do you consider this condition of the thumb CMC joint to be:
- A precursor to thumb CMC osteoarthritis
- A standalone condition/diagnosis

Which of the following terms do you commonly use to refer to this condition of the CMC-joint? (Select all that apply)
- ☐ Stage I thumb CMC arthritis (stage according to Eaton and Glickel[4])
- ☐ Early-stage thumb CMC arthritis
- ☐ Thumb CMC instability
- ☐ Other (Please specify): _______________

[if the participant chose multiple answers in the previous question]
Please specify in which context you use each term.

Answer: [FREE TEXT]

3. Frequency of Encounters:
We present a patient with non-traumatic pain of the thumb carpometacarpal (CMC) joint, with no chondral damage or total joint luxation on X-ray. Physical examination reveals excessive laxity of the CMC joint, particularly during dorsoradial displacement (see figure 1). The patient presents with no other joint instabilities.

How often do you encounter patients with the described condition in your practice?
- Daily
- Weekly

- Monthly
- Every few months
- Rarely
- Never

4. Physical Examination:
We present a patient with painful non-traumatic laxity of the thumb carpometacarpal (CMC) joint. The patient presents with no other joint instabilities. Please answer the following question as it applies to most patients in the general population. For this question, do not consider patients with unique circumstances that may prompt deviation from your standard of care.

If the patient has no chondral damage on X-ray, which physical examination finding(s) do you look for when diagnosing the described condition? (Select all that apply)

- ☐ Excessive laxity on dorsoradial displacement of the first metacarpal (see Figure 1)
- ☐ Pain on dorsoradial displacement of the first metacarpal (see Figure 1)
- ☐ Subluxation of the first metacarpal upon manipulation of the joint (see Figure 2)
- ☐ Tenderness upon palpation of the thumb CMC joint
- ☐ Other (please specify): _______________

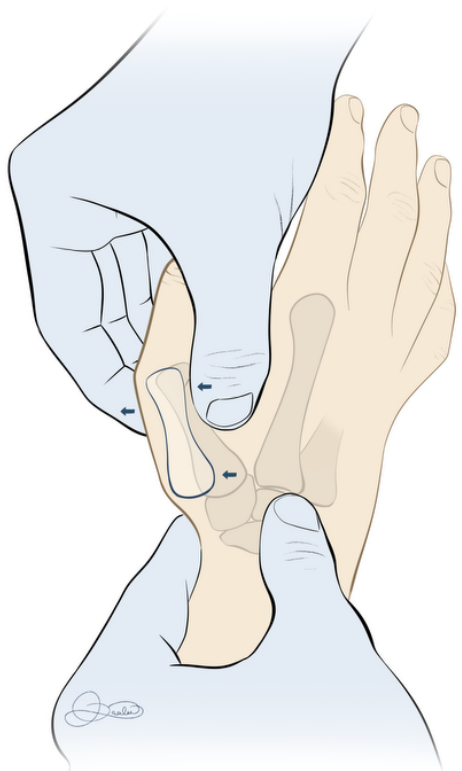

Figure 1: Dorsoradial displacement of the first metacarpal.

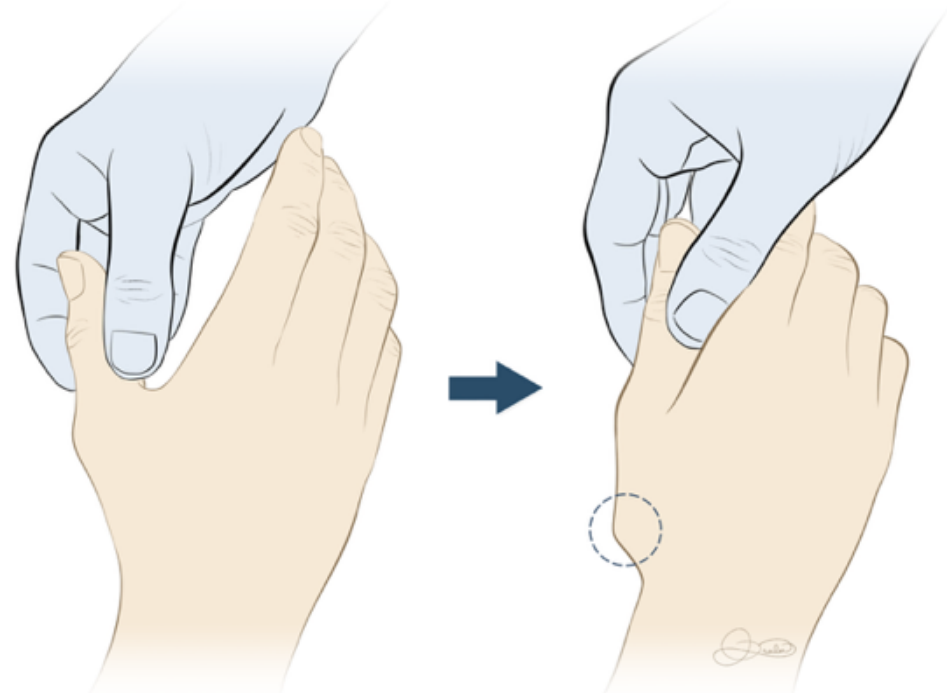

Figure 2: Visible subluxation of the first metacarpal bone on physical examination of the CMC joint.

Do you compare the laxity of the affected side to the contralateral side?
- Yes
- No

5. Diagnostic Workup:
We present a patient with painful non-traumatic laxity of the thumb carpometacarpal (CMC) joint. The patient presents with no other joint instabilities. Please answer the following questions as it applies to most patients in the general population. For these questions, do not consider patients with unique circumstances that may prompt deviation from your standard of care.

Which of the following diagnostic modalities do you routinely use in the diagnostic work-up of the described condition? (Select all that apply)

- ☐ X-rays for excluding chondral damage
- ☐ X-rays to assess instability
- ☐ CT (Computed Tomography) scan for excluding chondral damage
- ☐ CT (Computed Tomography) scan to assess instability
- ☐ Ultrasound for excluding chondral damage

- ☐ Ultrasound to assess instability
- ☐ MRI (Magnetic Resonance Imaging) scan for excluding chondral damage
- ☐ MRI (Magnetic Resonance Imaging) scan to assess instability
- ☐ Other (Please specify): _______________
- ☐ None

[if the participant chose any other answer, then only 'None' in the previous question]
[facultative question]
Please provide details on how you utilize this modality (e.g., stress views) and specify the findings you look for. Please provide a description for every chosen modality.

Answer: [FREE TEXT]

6. Initial Treatment:
We present a patient with painful non-traumatic laxity of the thumb carpometacarpal (CMC) joint, with no chondral damage or total joint luxation on X-ray. The patient presents with no other joint instabilities. Please answer the following questions as it applies to most patients in the general population. For these questions, do not consider patients with unique circumstances that may prompt deviation from your standard of care.

Which initial treatment plan would you propose for the described condition?
- ○ No treatment at all
- ○ Non-surgical measures (e.g. corticosteroid injections or splinting)
- ○ Surgical intervention

[if the participant chose 'Non-surgical measures (e.g. corticosteroid injections or splinting)' in the previous question]
Please specify which non-surgical measures you would propose.
- ☐ Corticosteroid injections
- ☐ Orthotic devices (e.g., thumb spica splint)
- ☐ Physical therapy
- ☐ Analgesics
- ☐ Other (Please specify): _______________

Please explain the reasons for your choice of initial treatment plan for the described condition
Answer: [FREE TEXT]

[if the participant chose 'Non-surgical measures (e.g. corticosteroid injections or splinting)' in the first question]
If the non-surgical measures prove ineffective for this patient, what course of action would you propose?
- ○ Continue non-surgical management (e.g., splinting, analgesics)
- ○ Surgical intervention

[if the participant chose 'Continue non-surgical management' in the previous question]
Please provide reasons for your choice and specify whether you would consider surgery for this patient at all. If surgery is being considered, please specify the circumstances under which you would recommend it.

Answer: [FREE TEXT]

[if the participant chose 'No treatment at all' in the first question]
Please provide reasons for your choice and specify whether you would consider non-surgical measures or surgical intervention for this patient at all. Please specify the circumstances in which you would recommend each option.

Answer: [FREE TEXT]

7. Patient Selection Criteria:
We present a patient with painful non-traumatic laxity of the thumb carpometacarpal (CMC) joint, with no chondral damage or total joint luxation on X-ray. The patient presents with no other joint instabilities. Please answer the following question as it applies to most patients in the general population. For this question, do not consider patients with unique circumstances that may prompt deviation from your standard of care.

*What factors influence your decision to **not** recommend surgical intervention for the described condition? (Select all that apply)*

- ☐ Severity of symptoms
- ☐ Patient's age
- ☐ Patient's activity level
- ☐ Chondral damage on radiographs
- ☐ Other radiographic findings (Please specify): _______________
- ☐ Failure of conservative management
- ☐ None
- ☐ Other (Please specify): _______________

8. Surgical Treatment:
We present a patient with painful non-traumatic laxity of the thumb carpometacarpal (CMC) joint, with no chondral damage or total joint luxation on X-ray. The patient presents with no other joint instabilities. Please answer the following questions as it applies to most patients in the general population. For these questions, do not consider patients with unique circumstances that may prompt deviation from your standard of care.

Which specific surgical technique is your initial preferred treatment choice in the surgical management of the described condition?

- ○ Eaton-Littler ligament reconstruction[1]
- ○ Other ligament reconstruction technique
- ○ Dorsoradial ligament tightening / capsulodesis[5]
- ○ Closing wedge osteotomy of the thumb metacarpal (Wilson osteotomy[6])
- ○ Denervation of the thumb CMC joint
- ○ Arthrodesis of the thumb CMC joint
- ○ Trapeziectomy
- ○ Trapeziectomy and ligament reconstruction and tendon interposition without drill hole in first metcarpal (e.g. Weilby[7])
- ○ Trapeziectomy and ligament reconstruction and tendon interposition with drill hole in first metcarpal (e.g. Burton – Pellegrini[8])
- ○ Thermal shrinkage of the joint capsule during arthroscopy
- ○ Other (Please specify): _______________
- ○ None

[if the participant chose any other option than 'None']
Which specific surgical technique is your preferred treatment choice if the first option fails in the surgical management of the described condition?

- ○ Eaton-Littler ligament reconstruction[1]
- ○ Other ligament reconstruction technique
- ○ Dorsoradial ligament tightening / capsulodesis[5]
- ○ Closing wedge osteotomy of the thumb metacarpal (Wilson osteotomy[6])
- ○ Denervation of the thumb CMC joint

- Arthrodesis of the thumb CMC joint
- Trapeziectomy
- Trapeziectomy and ligament reconstruction and tendon interposition without drill hole in first metcarpal (e.g. Weilby[7])
- Trapeziectomy and ligament reconstruction and tendon interposition with drill hole in first metcarpal (e.g. Burton – Pellegrini[8])
- Thermal shrinkage of the joint capsule during arthroscopy
- Other (Please specify): _______________
- None

[if the participant chose any other option than 'None']
Which specific surgical technique is your preferred treatment choice if the first and second option fails in the surgical management of the described condition?

- Eaton-Littler ligament reconstruction[1]
- Other ligament reconstruction technique
- Dorsoradial ligament tightening / capsulodesis[5]
- Closing wedge osteotomy of the thumb metacarpal (Wilson osteotomy[6])
- Denervation of the thumb CMC joint
- Arthrodesis of the thumb CMC joint
- Trapeziectomy
- Trapeziectomy and ligament reconstruction and tendon interposition without drill hole in first metcarpal (e.g. Weilby[7])
- Trapeziectomy and ligament reconstruction and tendon interposition with drill hole in first metcarpal (e.g. Burton – Pellegrini[8])
- Thermal shrinkage of the joint capsule during arthroscopy
- Other (Please specify): _______________
- None

[if the participant chose 'Other ligament reconstruction technique' in the previous questions]
Which specific 'Other ligament reconstruction technique' do you employ? Please describe the surgical technique in detail.

Answer: [FREE TEXT]

When deciding on the appropriate surgical technique for treating the described condition, which factors do you consider most important? (Select all that apply)

- ☐ Patient-related factors (e.g., age, comorbidities)
- ☐ Severity of instability
- ☐ Degree of pain
- ☐ Duration of symptoms
- ☐ Patient's occupation or daily activities
- ☐ Patient's overall health and fitness
- ☐ Patient's compliance with postoperative rehabilitation
- ☐ Availability of surgical equipment or resources
- ☐ Cost and insurance coverage
- ☐ Previous treatments tried
- ☐ Surgical experience and familiarity with techniques
- ☐ Patient preference
- ☐ Expected recovery time
- ☐ Long-term outcomes
- ☐ Recommendations from colleagues or clinical guidelines
- ☐ Evidence from recent studies or literature
- ☐ Institutional protocols or policies

- ☐ Risk of complications or adverse effects
- ☐ Patient's psychological status
- ☐ Potential for preserving joint function and mobility
- ☐ Other (please specify): _______________

10. Generalized laxity:
We present a patient with painful non-traumatic laxity of the thumb carpometacarpal (CMC) joint, with no chondral damage or total joint luxation on X-ray. Please answer the following question as it applies to most patients in the general population. For this question, do not consider patients with unique circumstances that may prompt deviation from your standard of care.

If the patient were to present with multiple joint instabilities (in the context of generalized ligament laxity or Hypermobile Ehlers-Danlos Syndrome), would you alter your previously described terminology and treatment approach? If so, please specify the modifications you would implement.

- o Yes (Please specify): _______________
- o No

11. Additional Comments: [facultative question]
We present a patient with painful non-traumatic laxity of the thumb carpometacarpal (CMC) joint, with no chondral damage or total joint luxation on X-ray.

Please share any additional thoughts, experiences, or considerations regarding the terminology, diagnosis, and management of the described condition.

Answer: [FREE TEXT]

End:
Thank you for completing the survey. Your contributions are greatly appreciated and will be instrumental in enhancing our understanding of this condition. Your insights will help inform future research and clinical practices, ultimately benefiting patients. If you have any additional comments or suggestions, please don't hesitate to share them with the survey creators via e-mail. Once again, we sincerely appreciate your time and valuable contributions.

References:

1. Eaton RG, Littler JW. Ligament reconstruction for the painful thumb carpometacarpal joint. JBJS. 1973;55(8).
2. Saccomanno MF, Fodale M, Capasso L, Cazzato G, Milano G. Generalized joint laxity and multidirectional instability of the shoulder. Joints. 2014;1(4):171-179.
3. Jongen IC, Nieuwdorp NJ, Hundepool CA, Van Gelder FS, Schutter AM, Zuidam JM. Ligament reconstruction in thumb carpometacarpal joint instability: A systematic review. JPRAS Open. 2024;39:237-248.
4. Eaton RG, Glickel SZ. Trapeziometacarpal osteoarthritis. staging as a rationale for treatment. Hand Clin. 1987;3(4):455-471.
5. Rayan G, Do V. Dorsoradial capsulodesis for trapeziometacarpal joint instability. *J Hand Surg Am*. 2013;38(2):382-387.
6. Wilson JN. Basal osteotomy of the first metacarpal in the treatment of arthritis of the carpometacarpal joint of the thumb. Br J Surg. 1973;60(11):854-858.
7. Weilby A. Tendon interposition arthroplasty of the first carpo-metacarpal joint. J Hand Surg Br. 1988;13(4):421-425.
8. Burton RI, Pellegrini VD Jr. Surgical management of basal joint arthritis of the thumb. Part II. Ligament reconstruction with tendon interposition arthroplasty. J Hand Surg Am. 1986;11(3):324-332.
