# Supplementary material for: Thumb Carpometacarpal Instability: An International Survey on Terminology, Diagnostic Workup, and Treatment
Source: Hand (N Y). 2026 Aug 19:15589447261469600. Online ahead of print. doi: 10.1177/15589447261469600 (PMC13490322; doi:10.1177/15589447261469600)
Supplement: Supplementary file 2 [file sj-pdf-2-han-10.1177_15589447261469600.pdf]

Supplemental Table S2:

Diagnostic modalities used in diagnosing thumb carpometacarpal instability.

Diagnostic modality		Number of surgeons (N, %)
X-ray	199 (96)	
For excluding chondral damage		187 (90)
For assessing instability		91 (44)
MRI	75 (36)	
For excluding chondral damage		59 (28)
For assessing instability		31 (15)
CT	27 (13)	
For excluding chondral damage		25 (12)
For assessing instability		3 (1)
Ultrasound	27 (13)	
For excluding chondral damage		25 (12)
For assessing instability		9 (4)
Other	9 (4)	
Arthroscopy		4 (2)
Fluoroscopy		4 (2)
Beighton-Scores		1 (0)
None	3 (1)	
