# Supplementary material for: Thumb Carpometacarpal Instability: An International Survey on Terminology, Diagnostic Workup, and Treatment
Source: Hand (N Y). 2026 Aug 19:15589447261469600. Online ahead of print. doi: 10.1177/15589447261469600 (PMC13490322; doi:10.1177/15589447261469600)
Supplement: Supplementary file 3 [file sj-pdf-3-han-10.1177_15589447261469600.pdf]

**Supplemental Table S3:**

Factors influencing the decision not to recommend surgical treatment for thumb CMC instability.

| **Diagnostic modality** | **Number of surgeons (N, %)** |
|---|---|
| Severity of symptoms | 128 (62) |
| Patient's age | 113 (54) |
| Patient's activity level | 99 (48) |
| Chondral damage on radiographs | 82 (39) |
| Failure of conservative management | 56 (27) |
| Other radiographic findings | 32 (15) |
| None | 21 (10) |
| Other | 13 (6) |
