# Supplementary material for: Thumb Carpometacarpal Instability: An International Survey on Terminology, Diagnostic Workup, and Treatment
Source: Hand (N Y). 2026 Aug 19:15589447261469600. Online ahead of print. doi: 10.1177/15589447261469600 (PMC13490322; doi:10.1177/15589447261469600)
Supplement: Supplementary file 4 [file sj-pdf-4-han-10.1177_15589447261469600.pdf]

Supplemental Material S4:

Sankey plot illustrating surgical techniques used for thumb CMC instability, including second- and third-line procedures after failure of initial surgical treatment.

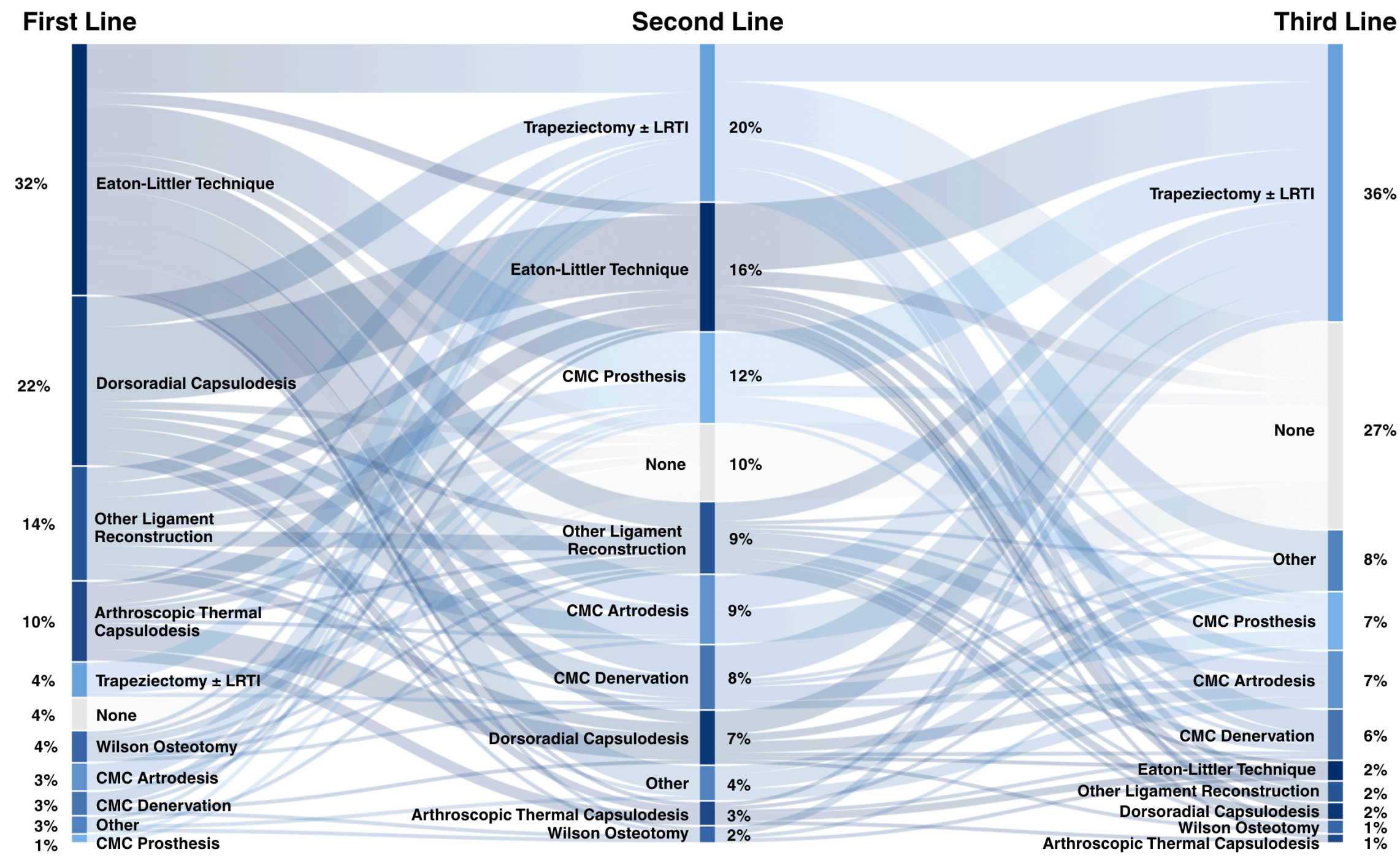

LRTI Ligament Reconstruction and Tendon Interposition.
