# Supplementary material for: Thumb Carpometacarpal Instability: An International Survey on Terminology, Diagnostic Workup, and Treatment
Source: Hand (N Y). 2026 Aug 19:15589447261469600. Online ahead of print. doi: 10.1177/15589447261469600 (PMC13490322; doi:10.1177/15589447261469600)
Supplement: Supplementary file 5 [file sj-pdf-5-han-10.1177_15589447261469600.pdf]

Supplemental Table S5

Factors considered most important when selecting a surgical technique for thumb CMC instability.

Term	Number of surgeons N (%)
Degree of pain	157 (76)
Severity of instability	143 (69)
Patient-related factors (age, comorbidities)	142 (69)
Patients' occupation or daily activities	141 (68)
Previous treatments tried	122 (59)
Patients' compliance with postoperative rehabilitation	91 (44)
Duration of symptoms	90 (43)
Potential for preserving joint function and mobility	88 (42)
Evidence from recent studies or literature	82 (39)
Patient preference	82 (39)
Surgical experience and familiarity with techniques	70 (34)
Patients' overall health and fitness	67 (32)
Long-term outcomes	65 (31)
Risk of complications or adverse effects	61 (29)
Patients' psychological status	60 (29)
Recommendations from colleagues or clinical guidelines	44 (21)
Expected recovery time	41 (20)
Availability of surgical equipment or resources	18 (9)
Cost and insurance coverage	12 (6)
Institutional protocols or policies	11 (5)
Presence of degenerative joint changes	1 (0)
Response to splinting	1 (0)
Radiographic features	1 (0)
