# Supplementary material for: Thumb Carpometacarpal Instability: An International Survey on Terminology, Diagnostic Workup, and Treatment
Source: Hand (N Y). 2026 Aug 19:15589447261469600. Online ahead of print. doi: 10.1177/15589447261469600 (PMC13490322; doi:10.1177/15589447261469600)

Supplemental Material S6:

Frequency of encountering thumb CMC instability stratified by geographic region. Surgeons in the United States reported higher encounter rates than those in Europe.

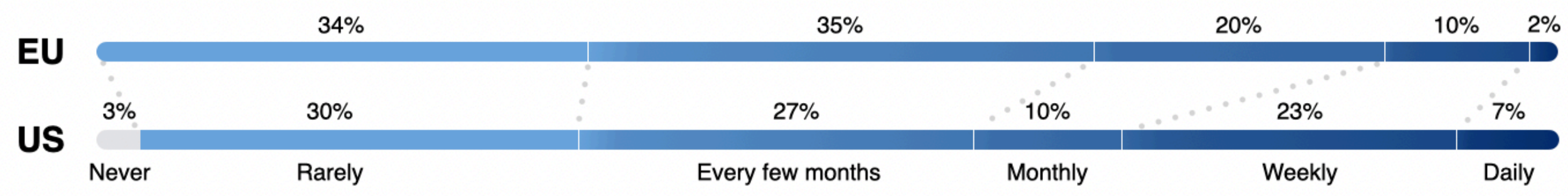

Supplement: Supplementary file 6 [file sj-pdf-6-han-10.1177_15589447261469600.pdf]
