# Supplementary material for: Thumb Carpometacarpal Instability: An International Survey on Terminology, Diagnostic Workup, and Treatment
Source: Hand (N Y). 2026 Aug 19:15589447261469600. Online ahead of print. doi: 10.1177/15589447261469600 (PMC13490322; doi:10.1177/15589447261469600)
Supplement: Supplementary file 7 [file sj-pdf-7-han-10.1177_15589447261469600.pdf]

Supplemental Material S7:

Preferred surgical techniques for thumb CMC instability stratified by specialty.

Initial surgical choice differed between plastic and orthopedic surgeons.

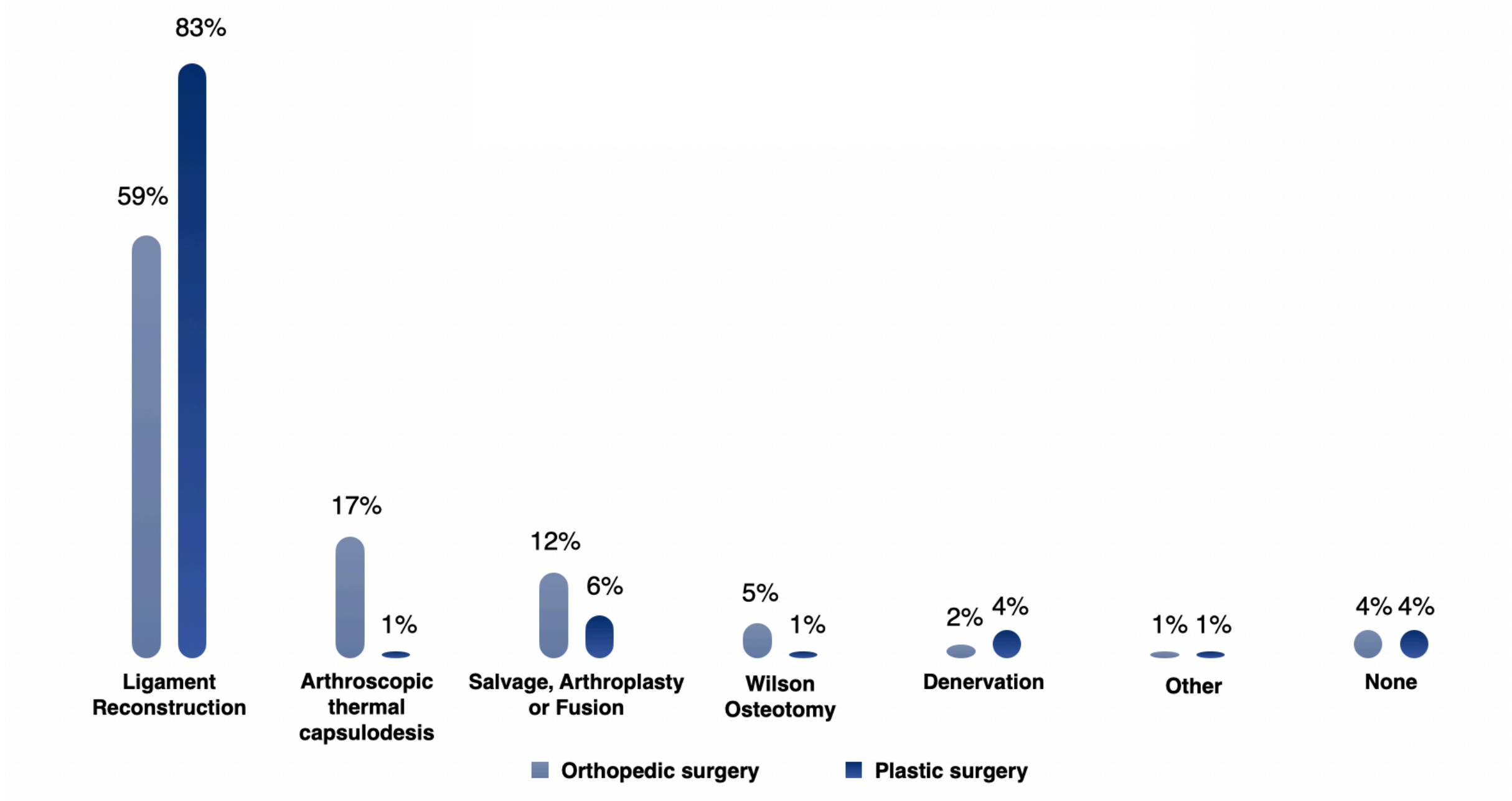

Ligament Reconstruction includes Eaton-Littler reconstruction, dorsoradial capsulodesis and other ligament reconstruction techniques. *Salvage, Arthroplasty or Fusion* includes trapeziectomy ± ligament reconstruction and tendon interposition, CMC prosthesis, and arthrodesis.
