# Supplementary material for: Thumb Carpometacarpal Instability: An International Survey on Terminology, Diagnostic Workup, and Treatment
Source: Hand (N Y). 2026 Aug 19:15589447261469600. Online ahead of print. doi: 10.1177/15589447261469600 (PMC13490322; doi:10.1177/15589447261469600)
Supplement: Supplementary file 8 [file sj-pdf-8-han-10.1177_15589447261469600.pdf]

Supplemental Material S1: Survey on terminology, diagnosis, and management of thumb CMC instability.

Supplemental Table S2: Diagnostic modalities used in diagnosing thumb carpometacarpal instability.

Supplemental Table S3: Factors influencing the decision not to recommend surgical treatment for thumb CMC instability.

Supplemental Material S4: Sankey plot illustrating surgical techniques used for thumb CMC instability, including second- and third-line procedures after failure of initial surgical treatment.

Supplemental Table S5: Factors considered most important when selecting a surgical technique for thumb CMC instability.

Supplemental Material S6: Frequency of encountering thumb CMC instability stratified by geographic region. Surgeons in the United States reported higher encounter rates than those in Europe.

Supplemental Material S7: Preferred surgical techniques for thumb CMC instability stratified by specialty. Initial surgical choice differed between plastic and orthopedic surgeons.
